# Omega-3 fatty acids: multi-target mechanisms and therapeutic applications in neurodevelopmental disorders and epilepsy

**DOI:** 10.3389/fnut.2025.1598588

**Published:** 2025-05-30

**Authors:** Miao Li, Zhiqiang Li, Yuying Fan

**Affiliations:** Department of Pediatrics, Shengjing Hospital of China Medical University, Shenyang, Liaoning, China

**Keywords:** Omega-3 fatty acid, hyperactivity disorder, autism spectrum disorder, tic disorder, epilepsy

## Abstract

Neurodevelopmental disorders (NDDs), including attention deficit hyperactivity disorder (ADHD), autism spectrum disorder (ASD), and Tourette's syndrome (TS), impair brain development and function, primarily affecting cognition, behavior, and social skills in children. Epilepsy, characterized by recurrent seizures due to neuronal hyperexcitability, shares pathological mechanisms with NDDs, such as neuroinflammation, synaptic dysfunction, and oxidative stress. Omega-3 fatty acids—primarily docosahexaenoic acid (DHA) and eicosapentaenoic acid (EPA)—exert neuroprotective and neuromodulatory effects in these conditions through multifaceted mechanisms. Omega-3 fatty acids play a role in activating the Nrf2/ARE pathway, protecting neurons from oxidative damage, regulating the gut-brain axis, and regulating the balance of microflora. Although Omega-3 fatty acids have a natural safety advantage in improving NDDs and epilepsy symptoms, the bioavailability is limited by the source, formulation form, and dietary environment. Current studies point out that monotherapy has a limited effect and requires a combination of vitamin D, probiotics, or drugs, as well as the development of innovative functional foods to improve intake efficiency. This review summarizes the multi-pathway roles of Omega-3 fatty acids in NDDs and epilepsy, emphasizing the potential as a core component of integrated treatment strategies. Future studies should prioritize precision nutrition approaches and functional food development to optimize patient outcomes in neuropsychiatric care.

## 1 Introduction

Neurodevelopmental disorders (NDDs) are a group of conditions impairing brain development and function, primarily affecting cognition, behavior, and social skills in children (1, 2). Common NDDs include attention deficit hyperactivity disorder (ADHD) (3), autism spectrum disorder (ASD) (4), and Tourette's syndrome (TS) (5), often linked to genetic, and epigenetic dysregulation (6). While epilepsy is classified as a neurological disorder, it shares mechanistic overlaps with NDDs, including neurodevelopmental origins and cortico-striatal circuit abnormalities (7). These parallels highlight potential shared therapeutic targets, such as synaptic dysfunction and neuroinflammation, which are critical for intervention strategies.

Omega-3 fatty acids constitute a class of polyunsaturated fats that a double bond at the third carbon at the methyl (omega) end of the molecule (8), known as the Omega-3 fatty acids position. The primary constituents of Omega-3 fatty acids are α-linolenic acid (ALA), along with its metabolites docosahexaenoic acid (DHA) and eicosatetraenoic acid (EPA). The growing interest in plant-derived natural compounds, such as curcumin (9), resveratrol (10), and quercetin, has underscored their potential in modulating neuroinflammation and oxidative stress. Plants offer potential benefits for improving ADHD symptoms by providing Omega-3 fatty acids, especially α-linolenic acid (ALA). Patients with ADHD may have deficiencies in essential fatty acids. Plant-derived ALA can be used as a source of Omega-3 fatty acids that the body cannot synthesize on its own, supplementing this deficiency and thereby improving neurodevelopment and function. As essential fats, they play a crucial role in the structure of cell membranes, nerve signaling, and anti-inflammatory responses through dietary supplementation (11). Marine organisms such as salmon (12), mackerel (13), and sardines (14) are rich in DHA and EPA, while plants such as flaxseed (15), chia seed (16), and walnut (17) are dominated by ALA.

As one of the main substances of Omega-3 fatty acids, DHA accounts for 20%−30% of brain lipids and 50%−60% of retinal lipids, mainly for neuroprotection (18, 19), through structural support, DHA plays multiple key roles in the nervous system: as a major component of neuronal cell membranes and myelin sheaths, DHA maintains membrane fluidity and ensures the proper functioning of synaptic functions, while promoting the synthesis and release of neurotransmitters such as acetylcholine and serotonin, thereby enhancing cognitive function (20), It also inhibits the production of pro-inflammatory mediators such as IL-6 and attenuates oxidative stress-related damage, including β-amyloid accumulation (19, 21). Studies have shown that DHA intake is effective in improving Alzheimer's disease (22, 23), depression (24) is effective and essential for infant brain development (25). Furthermore, research indicates that supplementing with omega-3 fatty acids during pregnancy may boost a child's cognitive growth, which is evident in higher intelligence quotient scores.

As one of the other primary components of Omega-3 fatty acids, EPA also has neuroprotective effects (26), but the two have different modes of action, and EPA mainly plays a role in cardiovascular and cerebrovascular aspects (27, 28). These benefits encompass anti-inflammatory regulation, including a decrease in the synthesis of pro-inflammatory eicosanoids, such as prostaglandin E2 by competitive inhibition of arachidonic acid metabolism (29, 30); improve cerebral blood flow: regulate vascular endothelial function and increase cerebral microcirculation (31); mood regulation: adjunctive treatment for depression and anxiety disorders (better at a 2:1 ≥ of apparent EPA:DHA in studies (32).

Omega-3 fatty acids, have become the treatment of NDDs due to their multi-target regulatory effects on the nervous system NDDs and epilepsy. This new type of intervention, such as natural substances such as Omega-3 fatty acids, has a higher safety profile and fewer side effects due to its natural origin. It has a wide range of physiological effects and can affect multiple biological pathways including cardiovascular, neurological, and immune systems. What's more, it is not limited to symptom relief, but may also show the potential to alter the course of the disease by modulating pathways such as neuroinflammation and oxidative stress, especially for neurodegenerative diseases. In addition, its multi-target mechanisms such as anti-inflammatory, promoting synaptic plasticity and antioxidant make it unique in improving diseases such as neurodevelopmental disorders and epilepsy, reducing inflammatory responses, enhancing neuronal connections, and scavenging free radicals, thereby improving patients' cognitive function and neurodevelopment in an all-round way. In the future, precision medicine strategies should be used to maximize the clinical benefits of molecular typing and individualized dosing. This overview will present the latest insights into the functions of DHA and EPA in NDDs and their application in the treatment of epilepsy, drawing on evidence from clinical research.

## 2 Neurodevelopmental disorders and features of epilepsy

ADHD, a prevalent neurodevelopmental disorder, impacts individuals across the globe, including children and adults, and is largely influenced by genetic factors (33), the main characteristics include inattention, hyperactivity, and impulsive behavior (34). This disorder can occur in both children and adults, affecting about 5% to 7% of children worldwide. ADHD is characterized by distraction and hyperactivity-impulsive tendencies, as well as distraction accompanied by excessive physical activity. Its onset not only affects concentration, but can also lead to learning difficulties. If left untreated, the symptoms of ADHD in childhood can persist into adulthood. Furthermore, individuals with ADHD might also encounter behavioral issues, learning disabilities, substance abuse, anxiety, depression, and other difficulties, which can have implications for their family dynamics, professional careers, and other aspects of their lives.

As one of the NDDs, ASD mainly affects people's socialization, communication, learning and behavior. This disorder is considered a developmental disorder and can be diagnosed at any age (35, 36). It occurs mainly in infancy and toddlerhood, with a predominance of 2–3-year-olds, and new research suggests that it may continue into school age (37). The type and severity of symptoms of ASD vary widely between individuals and can be lifelong and cause great distress to the family (38). ASD is marked by challenges in social communication and interaction, frequently accompanied by engaging in repetitive and routine behaviors, as well as having restricted and fixated interests and activities. The degree of impairment of these features is taken into account when diagnosing ASD to differentiate the diagnosis of typical autism (39), Asperger's syndrome (40), and individuals with pervasive developmental disorders to be classified.

TS, a chronic condition, is characterized by the presence of multiple motor and vocal tics across various body regions, with some individuals experiencing coprolalia or other abnormal vocalizations (41). This syndrome usually begins in childhood and may persist into adulthood. Symptoms include brief, rapid, and sudden involuntary movements, such as frequent blinking, brow-squeezing, sniffing, pouting, mouth opening, tongue sticking out, nodding, etc. (42). As the condition worsens, you may shrug your shoulders, twist your neck, shake your head, kick your legs, shake your hands, or jerk your limbs. These symptoms may be more pronounced during times of emotional stress or anxiety and disappear after falling asleep (43). In addition, TS patients may also have problems such as poor concentration or decreased grades in class, which brings great inconvenience to study and life. The etiology of TS remains incompletely elucidated; however, genetic factors are considered to play a significant role in its development. In addition, DA hyper conduction or DA receptor hypersensitivity in the central nervous system may be involved.

**Figure 1:**
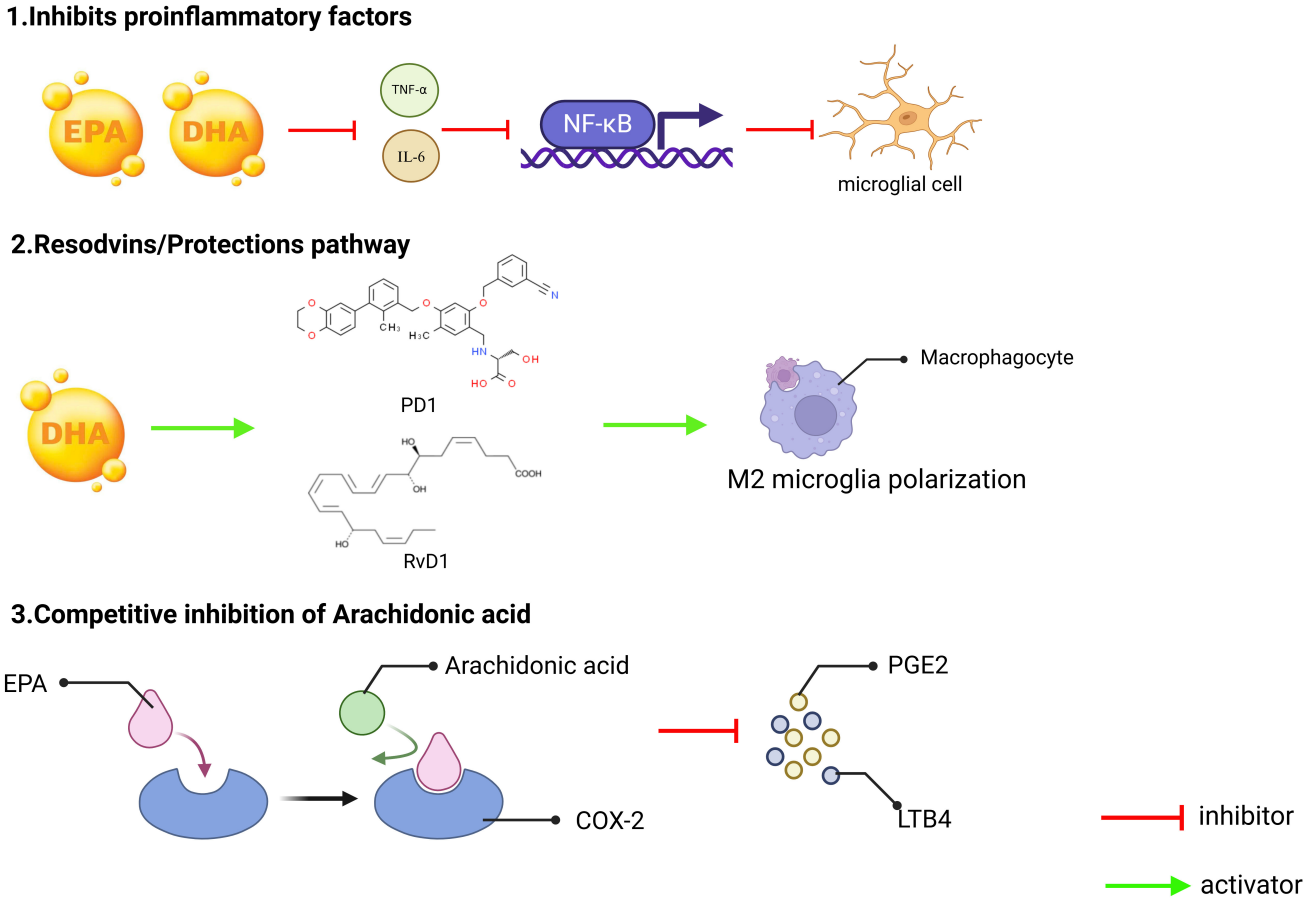
Interactive mechanisms of neuroinflammation, synaptic dysfunction, and oxidative stress in neurological disorders. (1) Neuroinflammation. TNF-α, IL-6, IL-1β promote the activation of NF-κB complexes and enter the nucleus, initiating pro-inflammatory gene expression and exacerbating neuroinflammation. As constituents of the central nervous system, microglia play a crucial role in their activation releasing pro-inflammatory factors, which further activate NF-κB and form a vicious circle of inflammation. At the same time, this circuit involves the connection between the cerebral cortex and the striatum, which may lead to impaired motor control and cognitive function; (2) Dyssynaptic function. Glutamate is the main excitatory neurotransmitter, but overactivation of N-Methyl-D-Aspartate (NMDA) receptors can lead to excessive influx of calcium ions, triggering neuronal damage or death (excitotoxicity), and NMDA receptor status will continue to open due to calcium overload, disrupting synaptic function, and the direct consequence of synaptic dysfunction and neuronal damage is manifested as impaired learning, memory and other higher brain functions. As a key postsynaptic scaffold protein, Post-Synaptic Density (PSD)-95 not only plays an important role in maintaining synaptic structure and function, but is also closely related to brain aging, neuropsychiatric diseases and brain injury repair; and (3) Oxidative stress. Inflammation and mitochondrial dysfunction lead to excessive accumulation of Reactive Oxygen Species (ROS), which decreases SOD and Mean Arterial Pressure (MDA), SOD-related free radicals are damaged by direct oxidation, and MDA destroys biomolecular structures mainly through cross-linking reactions, which in turn attack proteins, lipids, and DNA, exacerbate cell damage, and make mitochondrial production insufficient, resulting in insufficient neuronal energy supply and accelerating apoptosis. Under oxidative stress, β-amyloid (Aβ) aggregates abnormally, forming soluble oligomers and insoluble fibrous deposits β-amyloid plaques. These plaques are mainly deposited in extracellular areas of the brain and are one of the main pathological features of AD.

Epilepsy is a chronic brain disease caused by sudden, abnormal electrical discharges from neurons in the brain (44), which manifests as transient cerebral dysfunction. The disorder is characterized by recurrent and episodic attacks, in which patients experience multiple and brief episodes, each of which behaves similarly. Seizure symptoms are varied and may include momentary loss of consciousness and falls, paresthesia in the limbs, hallucinations, repetitive words or individual syllables, and rotation of the body or eyes. The causes of epilepsy may include disorders of cerebral cortical development, brain tumors, head trauma, central nervous system infections, etc., and may be related to genetics. The onset of epilepsy is relatively common in children and older adults (>65 years old) (45). According to statistics, epilepsy affects more than 70 million people worldwide (Table 1, Figure 1).

**Table 1:** Epidemiological characteristics of NDDs and epilepsy.

**Disease**	**Incidence/prevalence**	**Gender differences**	**Age of high incidence**	**Comorbidities and risk factors**	**References**
ADHD	Global prevalence in children is about 5%−7% Adult prevalence is approximately 2.5%−4%	Male: Female ≈ 3:1	School-age (6–12 years old)	Comorbidities: learning disability (30%), anxiety/depression (25%) Risk: preterm birth, low birth weight, hereditary (heritability ≈ 75%)	(33)
ASD	Global prevalence is approximately 1%−2%	Male: Female ≈ 4:1	Early childhood (2–3 years old)	Comorbidities: intellectual disability (30%), epilepsy (20%−30%) Risk: older parents, genetic mutations (e.g., SHANK3, NLGN3).	(36)
TS	The prevalence ranges from 0.3% to 1%, and about 50% of patients have comorbid obsessive-compulsive disorder (OCD) or ADHD	Male: Female ≈ 3-4:1	Childhood (5–10 years old)	Comorbidities: ADHD (60%), OCD (50%) Risk: basal ganglia dopamine dysfunction, heredity (SLITRK1 gene).	(43)
Epilepsy	The global prevalence is about 0.5%−1%, with the highest incidence in childhood (about 3%−5%)	There were no significant gender differences	Bimodal distribution (<5 years old, >65 years old)	Comorbidities: NDDs (e.g., ASD, intellectual disability) risk: brain injury, heredity (SCN1A gene), infection, hypoxia	(156)

## 3 Bioavailability and metabolic characteristics of Omega-3 fatty acids

### 3.1 Intake and absorption of Omega-3 fatty acids

Omega-3 fatty acids are critical to human health as they are indispensable nutrients that the body is unable to produce endogenously, necessitating their intake via the diet, and have a wide range of sources, such as natural foods such as deep-sea fish (salmon, mackerel, sardines), algae (schizolichytrid), and can be supplemented in a timely manner, in addition, it can also be supplemented in the form of supplements, such as fish oil, algae oil, etc. While marine-derived EPA and DHA are directly bioavailable, plant-based ALA requires enzymatic conversion to its active forms. However, emerging encapsulation technologies (e.g., nanoemulsions (46) of flaxseed oil) can enhance ALA bioavailability, mirroring advancements in delivering other plant compounds like curcumin and Epigallocatechin-3-gallate (EGCG).

Omega-3 fatty acids, once consumed by humans, must cross the blood-brain barrier (BBB) to be effectively utilized by the body. This process of traversing the BBB occurs through two distinct mechanisms: one of which is carrier-mediated transport (47). Lysine phosphatidylcholine (LPC), as a carrier of DHA, exerts its important neuroprotective role by transporting DHA into the brain through the mediation of the Mfsd2a protein, targeting LPC-DHA to transport into the brain parenchyma (48), which offers great potential to improve brain health, but the competitive inhibition of excess saturated fatty acids inhibits the transport function of Mfsa2a and reduces the intake rate of DHA (21); Second, through specific enrichment of brain regions (49), so that the concentration of DHA in the hippocampus and prefrontal cortex reached the highest (50).

### 3.2 Metabolic pathways of Omega-3 fatty acids

#### 3.2.1 Enzymatic reactions and metabolites

The metabolism of Omega-3 fatty acids involves several key pathways, including the cyclooxygenase (COX) pathway, the lipoxygenase (LOX) pathway, and the cytochrome P450 (CYP450) pathway. These pathways contribute to the conversion of Omega-3 fatty acids into various bioactive metabolites that exert diverse physiological effects. Studies have shown that EPA and DHA, when metabolized by the COX pathway, regulate the activity of COX-2, which in turn promotes the production of E-series mediators (RvE1) and D-series parsens (RvD1) (50) and the D-series resolvins (RvD1) (51) Thus allowing Omega-3 fatty acids to produce anti-inflammatory mediators that regulate the expression and activity of COX-2 to influence the inflammatory process (52). The LOX pathway curbs inflammation and fosters neuronal viability through the generation of LOX lipid mediators, particularly significant in major depressive disorders and hippocampal neurogenesis in humans, through 15-LOX enzyme catalytic production of protectin (PD1) and hippuric acid (MaR1) (53). The cytochrome P450 pathway refers to the cardioprotective effects of EPA and DHA as effective replacement substrates for the metabolism of cytochrome P450 (CYP) enzymes in arachidonic acid (AA) (54), which simultaneously regulates vasodilation and cerebral blood flow (55). Omega-3 fatty acids have been shown to activate the Nrf2/ARE signaling pathway (56, 57), which is a key transcriptional regulator of cellular antioxidant defense mechanisms. This activation likely occurs through interactions with Keap1, the inhibitor of Nrf2 (58). By modulating Keap1, Omega-3 fatty acids can lead to the release and nuclear translocation of Nrf2. Once in the nucleus, Nrf2 binds to the antioxidant response element (ARE) sequence in the promoter regions of target genes, thereby upregulating the expression of a variety of antioxidant enzymes and phase II detoxification enzymes. These include superoxide dismutase (SOD), catalase (CAT), glutathione peroxidase (GPx), and heme oxygenase-1 (HO-1), among others. The increased expression of these enzymes enhances the cells' ability to scavenge reactive oxygen species (ROS) and reduce oxidative stress, ultimately protecting neurons from oxidative damage (59).

#### 3.2.2 Core functions of anti-inflammatory mediators

Omega-3 fatty acids utilize the COX pathway and the LOX pathway, and their actions are mediated through specific compounds, primarily RvD1, PD1, and Neuroprotectins. RvD1 are derived from the conversion of EPA and are predominantly known for their anti-inflammatory effects, playing a central role in resolving inflammation with key functions: (1) inhibiting neutrophil infiltration to control the spread of inflammation, (2) promoting macrophages to phagocytosis apoptotic cells to help inflammation regress, and (3) terminating inflammatory responses (60, 61). PD1 are mainly converted by DHA and have anti-inflammatory, neuroprotective, and anti-apoptotic (anti-cell death) properties (62), Core functions: (1) Minimize oxidative stress and safeguard cells against harm, (2) reduce apoptosis, especially in nerve cells, protect hippocampal neurons from status epilepticus damage; neuroprotectins are converted by DHA, and the most well-known of these roles is neuroprotective hormin D1 (NPD1) (63), mainly inhibits β-amyloid toxicity: plays a role in Alzheimer's disease, specifically DHA-derived PD1, which protects nerve cells from damage by inhibiting β-amyloid toxicity (64).

#### 3.2.3 Individual differences in metabolic regulation

While the metabolic routes of Omega-3 fatty acids are multifaceted, they can be disrupted by both genetic and environmental influences, so that their regulatory mechanisms vary from individual to individual, mainly in two aspects, one is genetic factors, *FADS1/2* gene polymorphisms affect the conversion efficiency of Omega-3 fatty acids to long-chain fatty acids, and some people need to directly supplement DHA/EPA (65); Second, dietary interference, competitively inhibits Omega-3 fatty acids metabolism and weakens anti-inflammatory effects (66). At the same time, various factors affect the utilization of Omega-3 fatty acids, particularly when consumed as supplements or through dietary planning, individual differences, oxidative stability, etc., and the influencing mechanisms are also different (Table 2). The bioavailability of Omega-3 fatty acids is influenced by a combination of source form, metabolic enzyme activity, and dietary environment, and its metabolites exert multi-target effects through anti-inflammatory, antioxidant, and neuroprotective mechanisms. Optimizing intake strategies can maximize health benefits, especially for interventions in NDDs and epilepsy.

**Table 2:** Key factors influencing bioavailability.

**Factor**	**Influence mechanism**	**Optimize your strategy**	**References**
Supplement form	The TG form is 30%−50% more absorbent than the EE form and is less likely to cause gastrointestinal upset.	Choose TG or phospholipid-bound Omega-3 fatty acids (e.g., krill oil).	(157)
Meal synergy	Taking with a fat-containing meal increases absorption (bile secretion promotes emulsification).	Take with a meal with a food rich in monounsaturated fatty acids (such as avocado).	(158)
Individual metabolic capacity	Older adults, patients with liver disease, or those with FADS1/2 mutations have a low ability to convert to ALA.	Direct supplement pre-formed DHA/EPA (≥500 mg/d)	(159)
Oxidation stability	Fish oil is easy to oxidize to form aldehyde toxic substances, which reduces biological activity.	Choose sealed capsules containing antioxidants (vitamin E) and store them refrigerated.	(160, 161)

## 4 Molecular mechanisms by which Omega-3 fatty acids regulate neurological diseases

### 4.1 Neuroinflammation and immune regulation

Neuroinflammation represents the immune system's reaction to neural tissue, including the body's response to infection, trauma, or autoimmune diseases. The management of NDDs by Omega-3 fatty acids often entails the stimulation of immune cells and the modulation of inflammatory cytokine release (67). A particular study observed a notable rise in plasma DHA levels and the percentage of DHA in red blood cells among children with ASD who were given a DHA-enriched product. This investigation all diagnosed with ASD, and they were supplemented with the DHA-rich product for a duration of 6 months (68). Furthermore, research has explored the role of Omega-3 fatty acids polyunsaturated fatty acids on the cell surface in dampening the inflammatory reaction following experimental traumatic brain injury. In connection with this, the supplementation of Omega-3 fatty acids polyunsaturated fatty acids has been observed to reduce microglia-mediated inflammation following experimental traumatic brain injury by blocking the HMGB1/TLR4 pathway (69). These signaling pathways block the neuroinflammatory cascade by reducing the release of pro-inflammatory factors. In addition, 50 μM DPA significantly reduced BV2 cell viability after stimulation with 100 ng/mL LPS, demonstrating that n-3 DPA may inhibit microglia-NF-κB and MAPK p38 by balancing microglia M1 and M2 polarization (70). Another study showed unique pharmacological properties in which surface EDP-EA and EEQ-EAs dose-dependently decreased pro-inflammatory IL-6 cytokines and increased anti-inflammatory IL-10 cytokines (71).

**Figure 2:**
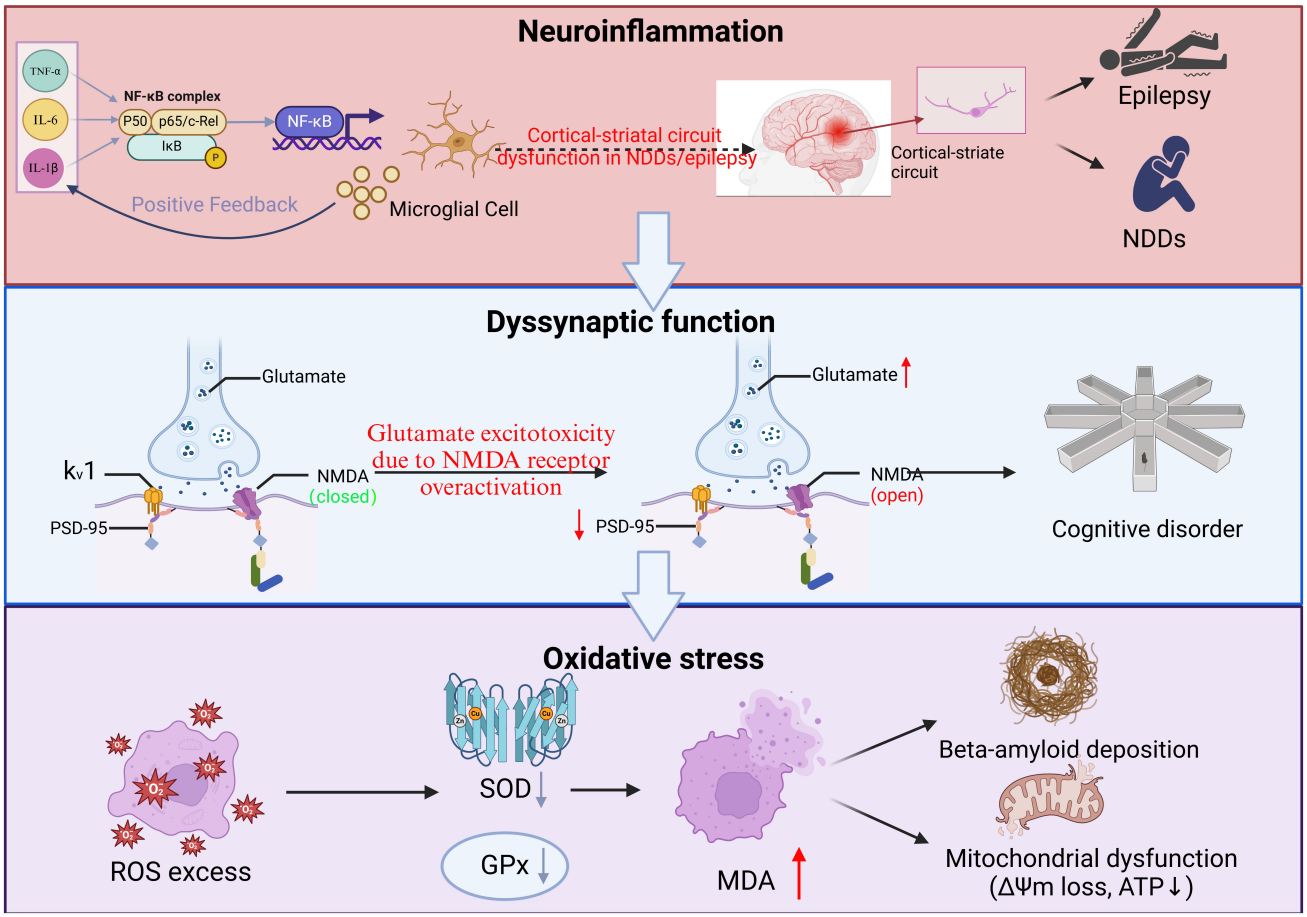
Three key mechanisms underlying the anti-inflammatory effects of omega-3 fatty acids (EPA and DHA). (1) Inhibition of pro-inflammatory factors. EPA and DHA can reduce the production of pro-inflammatory factors such as TNF-α and IL-6. These pro-inflammatory factors typically promote the inflammatory response by activating NF-κB, and by inhibiting the release of these pro-inflammatory factors, EPA and DHA can effectively reduce the degree of microglial activation, thereby reducing inflammation; (2) Conservation/Restoration Pathways. DHA can exert its anti-inflammatory effects by converting to protectins, such as PD1 and RvD1, which can induce macrophages to M2-type polarization. M2 macrophages are a subset of macrophages with immunomodulatory functions that contribute to tissue repair and resolution of inflammation; and (3) The metabolism of arachidonic acid is subject to competitive inhibition. Arachidonic acid can be metabolized in the body by COX-2 enzyme to generate PGE2 and LTB4, both known for their potent pro-inflammatory effects. EPA, an Omega-3 fatty acids, plays a role in this process, as an Omega-3 polyunsaturated fatty acid, can compete with arachidonic acid for the same enzyme sites or metabolic pathways, thereby reducing the production of PGE2 and LTB4, and in this way, EPA can indirectly inhibit the occurrence and progression of inflammatory responses.

Omega-3 fatty acids can competitively inhibit AA metabolism, and compete with AA to bind COX-2 and LOX enzymes through EPA, reducing the production of pro-inflammatory mediators' prostaglandin E2 and leukotriene B4 (72). At the same time, the generation of anti-inflammatory mediators through RvD1 and PD1 can also inhibit neuronal apoptosis and reduce oxidative stress (Figure 2).

### 4.2 Synaptic plasticity and neurotransmission

PSD-95, a protein in neurons with a PDZ domain, has been observed to promote the maturation of glutamatergic synapses in hippocampal neurons when its expression is increased (73), DHA promotes the expression of PSD-95 protein in the postsynaptic dense region and stabilizes glutamate receptors (such as NMDA receptors) (74). DHA also promotes synaptogenesis and synaptic expression in neurons, including the Synapsing protein, as well as the independent and co-cerebral effects of glutamate receptors in rat hippocampal neurons (75). Concurrently, research has demonstrated that the function of Omega-3 fatty acids within the nervous system is linked to their selective concentration in synapses, where they play a crucial role in synaptic structure and function, dendrites and photoreceptors, but their specific effects on synaptic function and plasticity have not been fully understood (76). It is hypothesized that DHA enhances long-term enhancement (LTP) in the hippocampus and improves learning and memory function by activating CaMKII and CREB signaling (77), This enhances long-term enhancement LTP (78). One study showed that Omega-3 fatty acids activated Akt phosphorylation, promoted mTOR-mediated protein synthesis, and supported neuronal survival and synaptic reconstruction (79). In a related study focusing on this pathway, research revealed that the intake of Omega-3 fatty acids polyunsaturated fatty acids boosted autophagy by repressing the activation of the PI3K/mTOR signaling pathway, leading to enhanced cognitive performance in heat-stressed mice, Heat stress upregulates phosphorylation of the PI3K-Akt-mTOR pathway in mouse hippocampus and HT22 cells. In contrast, omega-3 PUFA uptake significantly reduced phosphorylation levels within this pathway, alleviated the autophagic fusion barrier imposed by heat stress, and promoted autophagic flux (80). This indicates that Omega-3 fatty acids have a significant role in modulating this signaling pathway, which is instrumental in supporting the viability and functionality of neurons.

Modulation of the neurotransmitter system is a key process for maintaining the normal function of the nervous system (81). Excitatory amino acid transporters (EAATs) are crucial for preserving reduced levels of extracellular glutamate and for guarding against excitotoxicity (82). While DHA can reduce glutamate concentrations in the protruding interspace, inhibit AMPAR/NMDAR overactivation, reduce excitotoxicity, and study surfaces, targeting EAAT2 could be a promising approach for developing epilepsy therapies (83). DHA can also enhance the stability of β-catenin, promote neural stem cell differentiation and synapse formation, and repair neurodevelopmental defects (84, 85). In addition, Omega-3 fatty acids are able to upregulate the expression of γ-aminobutyric acid synthetase (86). By upregulating its expression, it can promote inhibitory signaling (87).

### 4.3 Apoptosis and autophagy regulation

Apoptosis is an integral part of organism's development, tissue remodeling, and disease treatment (88). Mitochondrial apoptosis also plays an indispensable role in this, and Omega-3 fatty acids can inhibit the mitochondrial apoptosis pathway, DHA upregulates the anti-apoptotic protein Bcl-2, inhibits the translocation of the pro-apoptotic protein Bax to mitochondria, and prevents the release of cytochrome C (89, 90). At the same time, caspase-9 activated by mitochondrial apoptosis further activates other caspases, leading to the disintegration of cellular structures and cell death, while Omega-3 fatty acids can reduce caspase-3 cleavage and protect neurons from apoptosis induced by β-amyloid or status epilepticus (91). In addition, DHEA and EPEA treatment induces phosphorylation of Bcl-2 and promotes its dissociation from beclin-1, resulting in autophagy induction, exerting antiproliferative effects (92).

Autophagy is a process of intracellular degradation and recycling of damaged or excess cellular components (93). Autophagy can remove abnormal proteins and restore the body's health, and Omega-3 fatty acids mainly play a role in EPA and DHA. mTORC1 directly inhibits AMP kinase (AMPK) activity, thereby promoting cell proliferation even under conditions of nutrient stress (94). The control effect of EPA can inhibit the mTORC1 complex and relieve its inhibitory effect on autophagy (95). DHA activates the potential role of the AMPK pathway in cytoprotective and metabolic regulation, and one study showed that DHA activates the protective effect of AMPK against retinal ischemic injury (96). During the growth of muscle fibers in sturgeon, the activation of the AMPK/Sirt1 pathway by DHA fosters muscle development (97). Specifically, by augmenting autophagy flux with DHA, an Omega-3 fatty acids polyunsaturated fatty acid. The activation of AMPK and the subsequent inhibition of mTOR can promote autophagy, which is a cellular process that helps to clear damaged proteins and organelles, thereby potentially protecting cardiac cells and improving heart function after injury (98). All of these mechanisms ultimately point to the formation of autophagosomes, which remove tau protein or α-synuclein aggregates, which play a significant role in the protection of Parkinson's disease, myocardial infarction, or retina.

Omega-3 fatty acids, especially DHA, inhibit apoptosis by upregulating Bcl-2 and preventing Bax translocation, while EPA enhances autophagic flux by inhibiting the mTORC1 complex. Their combined effects prevent excessive cell death and promote controlled degradation of damaged cellular components (69). The neuroprotective effect of omega-3 PUFA supplementation on TBI rats has been studied, and the direct interaction between cytoplasmic Beclin-1 and Bcl-2 induced by increasing SIRT1 activity has been studied, demonstrating the link between autophagy and apoptosis (99). This balance is vital in neurodegenerative diseases, where apoptosis and autophagy are dysregulated. Further research is needed to develop targeted interventions that maximize the therapeutic potential of Omega-3 fatty acids in neurological disorders.

### 4.4 Oxidative stress and mitochondrial function

Oxidative stress (OS) denotes the potential harm to a cell's architecture and functionality caused by the generation and build-up of oxidizing agents, such as ROS, within and outside the cell. This accumulation surpasses the capacity of the cell's antioxidant defenses to cope. To combat oxidative stress, most organisms boost the activity of enzymes like superoxide dismutase (SOD) and glutathione peroxidase, or they work to decrease the concentration of the lipid breakdown product malondialdehyde (MDA) (100, 101). To explore the underlying pathophysiological mechanisms of increased production of surface reactive oxygen species in AD, HD, and amyotrophic lateral sclerosis (102). In a study on the effects of Omega-3 fatty acids on the antioxidant enzyme system in skeletal muscle cells, supplementation with raw spinach or NBS superfoods for 7 days appeared to have a positive effect on the inflammatory response to repetitive anaerobic exertional activity (SOD, MDA, and IL-6) (103). Sows nourished with a diet deficient in Omega-3 polyunsaturated fatty acids, particularly low in ALA, EPA, and DHA, around the time of weaning, exhibited beneficial outcomes for their brain development, liver, and uterine lipid peroxidation and antioxidant enzyme activity. Elderly (24 months old) Wistar rats fed daily doses of 30 mg EPA showed beneficial effects of Omega-3 fatty acid supplementation on brain tissue and increased SOD and reduces lipid peroxidation (104). Supplementation with Omega-3 fatty acids (FAs) along with vitamin E has been shown to elevate levels of nitric oxide (NO) and total antioxidant capacity, while reducing levels of MDA, thereby providing further evidence of the impact of Omega-3 fatty acids on OS.

The main functions of mitochondria are currently shown to include: energy production, ROS production, calcium homeostasis and cell death, which play an indispensable role in energy metabolism. Omega-3s have been shown to have a positive effect on mitochondrial function optimization. Perilla seed oil is rich in Omega-3 fatty acids, manifested by decreased ROS levels, stable mitochondrial membrane potential, and increased ATP levels, provides healthy activity for the brain (105). In addition, DHA can also activate PGC-1α, promote mitochondrial DNA replication and respiratory chain complex expression, which plays an important role in biosynthesis (106). Moreover, in terms of ability to metabolize, Omega-3 fatty acids is an unobtainable existence to alleviate energy crises in epilepsy or neurodegenerative diseases.

### 4.5 Gut microbiome-brain axis regulation

**Figure 3:**
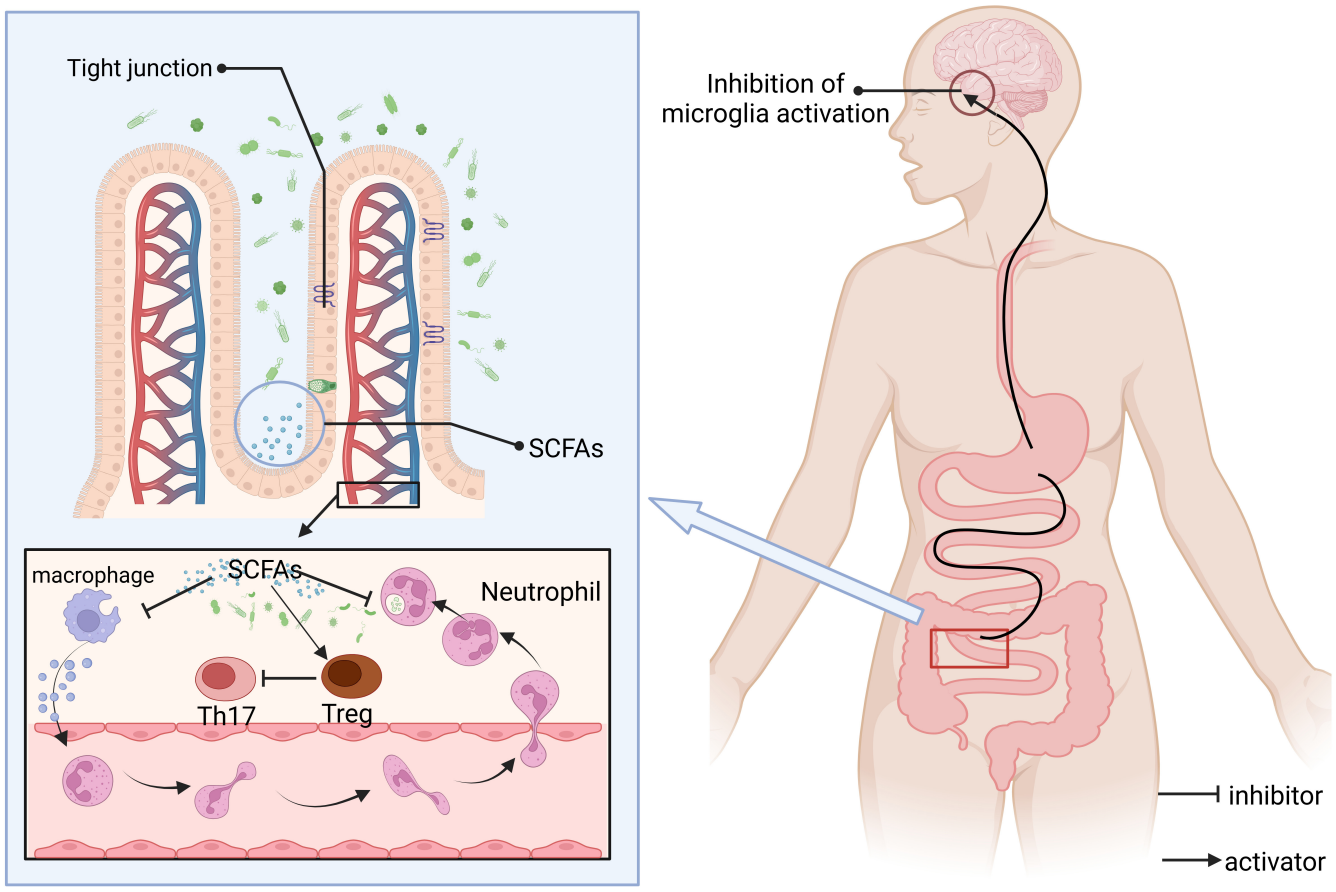
SCFAs regulate immune-neural interactions and barrier functions. SCFAs are produced by intestinal microbial fermentation of dietary fiber, which can reach the whole body through blood circulation and act as natural inhibitors, (1) inhibition of pro-inflammatory pathways: inhibition of microglial overactivation (reducing neuroinflammation); (2) Regulate immune homeostasis: promote the differentiation of anti-inflammatory regulatory Tregs and inhibit the activity of pro-inflammatory Th17 cells; and (3) Enhance the expression of tight junction proteins between epithelial cells, maintain barrier function, and prevent pathogens or toxins from leaking and causing inflammation. SCFAs may alleviate disease progression such as multiple sclerosis by inhibiting the Th17 pathway. Consuming a certain amount of Omega-3 fatty acids can boost the level of SCFAs.

The regulation of the gut microbiota-brain axis constitutes a reciprocal communication network. This interaction is mediated through the pathways of the nervous, immune, endocrine, and metabolic systems, playing a pivotal role in modulating states of anxiety, depression, and cognitive abilities, also impacts the initiation and advancement of neurodegenerative disorders, including Alzheimer's (107) and Parkinson's diseases (108), along with other pathological states (Figure 3). Inflammatory and immune diseases (colitis) play an unobtainable role. In one study related to AD, Omega-3 FA supplementation influenced the transition from gut microbiota balance to a symbiotic state, but the results were not significant enough (109). Research into the link between Omega-3 fatty acids and the gut-brain axis revealed that these fatty acids influence the makeup and operations of the gut microbiota, significantly boosting the prevalence of advantageous bacterial species (such as Bifidobacteria), which contributes to gut health and improves systemic immunity (110). Omega-3 PUFA supplementation was also observed to reduce the number of faecalibacterium, while the number of coecalibacterium and butyrate-producing bacteria in the Triocillaceae family increased, simultaneously, it fosters the generation of anti-inflammatory agents, including short-chain fatty acids (111). In addition, low Omega-3 PUFA intake can have deleterious effects on neurodevelopment, have long-term effects on behavior, and worsen early intestinal dysfunction induced by maternal immune activation (MIA) (112). The microbiota metabolizes Omega-3-generated SCFAs (e.g., butyric acid) to activate intestinal FFAR3 receptors that transmit anti-inflammatory signals to the brain via the vague nerve (113). Omega-3 polyunsaturated fatty acids are partially metabolized by anaerobic bacteria in the distal gut (e.g., *Bifidobacteria* and *Lactobacilli*), thereby affecting the distribution of gut microbiota, and dietary addition of omega-3 polyunsaturated fatty acids increases the abundance and percentage of bifidobacteria in the gut of male Sprague-Dawley rats (114). It may also benefit the number of lactic acid-producing bacteria and *bifidobacteria* (115). At the same time, the balance of intestinal microbes can be directly or indirectly changed (116). For example, after supplementation with Omega-3 PUFA, the abundance of Escherichia, *Faecalibacterium* and *Veronella* was detected in the IBD group, while the abundance of *Bacteroides* and *Flavobacterium* was decreased (117, 118). Moreover, SCFAs can promote immune homeostasis, regulatory T cell (Treg) differentiation, and inhibit central nervous inflammation (such as multiple sclerosis) (119). *E. coli* counts in high-fat mice after 12 weeks of supplementation with Omega 3 (200 mg/kg) and cannabidiol (CBD) showed that E. coli numbers were overgrown, suggesting that the synergistic effect of the two can improve HFD-induced anxiety and memory impairment (120).

## 5 Regulatory mechanisms of Omega-3 fatty acids for specific diseases

### 5.1 Attention deficit hyperactivity disorder

ADHD poses a significant challenge for children. This condition is often linked to enduring difficulties in social interactions, educational performance, and overall mental wellbeing. Omega-3 fatty acids is an essential fatty acid for the human body, which is easily obtained through food and other means, and has efficacy for a variety of diseases in terms of NDDs, and previous studies have shown that Omega-3 polyunsaturated fatty acids (PUFAs), particularly EPA and DHA, have been studied for their potential effectiveness in alleviating symptoms of ADHD. Some research suggests that Omega-3 fatty acids supplementation may improve certain symptoms of ADHD, such as inattention, hyperactivity, although the results are not consistently positive across all studies. More research is needed to establish the precise role of Omega-3 fatty acids PUFAs in ADHD treatment (91). A study of 1,789 patients found that neither high EPA doses nor high EPA/DHA ratios were found to improve the core symptoms of ADHD, When omega-3/omega-6 polyunsaturated fatty acid supplements were taken at doses of 558 mg EPA, 174 mg DHA, and 60 mg GLA (9:3:1 ratio) for 12 months, red blood cell levels were improved and adherence to medications such as methylphenidate (MPH) was improved (121), suggesting that treatment for ADHD may be a potential, long-term aspect. γ-linolenic acid (GLA), as an emerging fatty acid, may function as a co-anti-inflammatory agent (122). In a single and crossover trial involving 2,374 participants, the results of Omega-3 PUFA plus atomoxetine was compared with atomoxetine and Omega-3 or Omega-6 supplementation plus methylphenidate-methylphenidate, and the results of the two trials found that low-quality PUFAs may improve ADHD symptoms in the medium term, but there was no difference with higher doses compared to placebo (123).

Although the efficacy of Omega-3 fatty acids, especially EPA and DHA, for ADHD is controversial, some research suggests that Omega-3 supplements may improve certain symptoms. Differences in efficacy may be related to dose, fatty acid type (ALA, EPA, DHA), individual differences (e.g., FADS1/2 gene polymorphisms) (124), and differences in assessment methods. The anti-inflammatory effects of Omega-3 fatty acids, the improvement of neurotransmitter function, and the promoting effects on brain development may be its potential therapeutic mechanisms. Larger, more rigorous studies are needed in the future to further explore efficacy and mechanisms, and to develop personalized treatment plans. ADHD is a high-incidence disorder in school-age children and adolescents, and some evidence has been shown for the positive effects of Omega-3/Omega-6 fatty acids through parent-rated internalizing symptoms and parent- and teacher-rated externalizing symptoms (125). Research has also examined the inverse relationship between pronounced DHA/EPA deficiencies and the expression of ADHD symptoms, encompassing the involvement of various biological systems. This includes the role of inflammation, as well as imbalances in the gut-microbiota axis (GBA) (126). Based on this study, supplementation with a single Omega-3 PUFA therapy in adolescents improved the clinical symptoms and cognitive performance of children and adolescents with ADHD, further confirming that Omega-3 fatty acid deficiency may lead to exacerbation of symptoms in ADHD patients (127). In 179 children with low IQ or ADHD who received DHA/EPA-rich eggs for 3 months, the intake of Omega-3 PUFAs through dietary supplements enhanced visual clarity and altered red blood cell fatty acid compositions in school-aged children with below-average IQ or ADHD, showing a favorable comparison to those who ate standard eggs (128). Concurrently, the intake of supplements containing long-chain Omega-3 polyunsaturated fatty acids, such as EPA/DHA, has been shown to elevate the levels of EPA/DHA within red blood cell membranes and enhance working memory capabilities (129). In a study of 40 boys with ADHD who received 650 mg of EPA/DHA or placebo per day, the results showed that children receiving EPA/DHA supplementation had higher levels of phospholipid DHA at follow-up than placebo in children receiving EPA/DHA supplementation, thus supporting that Omega-3 supplementation may be an effective addition to ADHD drug therapy (130).

### 5.2 Autism spectrum disorder

ASD mostly occurs in infants and young children after the age of 2 years, and is mostly manifested as social communication deficits and the existence of limited, repetitive behaviors or interests, which are improved by the commonly used drugs risperidone and aripiprazole, while ASD patients have a higher probability of suffering from depression, anxiety, sleep difficulties, epilepsy, and intellectual disability (2). In a double-blind, involving 54 children with autism, those who received one 1,000 mg Omega-3 fatty acids capsule per day for an 8-week period showed improvements in autism-related characteristics. Significant improvements in stereotypical behaviors and social interaction were observed in the children who received Omega-3 supplementation, in contrast to those in the control group who were not given the Omega-3 supplement. Studies have shown that preterm children are more likely to develop ASD, and 31 children aged 18–38 months (children <29 weeks) were tested with Omega 3-6-9 or canola oil placebo, and Omega 3 had some improvement in those children who showed the usual early symptoms of ASD, but there may be individual differences due to the small sample size may not be sufficient to demonstrate significant improvement (131). In another sensory test for ASD in premature infants, which tests the senses of smell, sight, touch, etc., supplementation with Omega-3 fatty acids may enhance the sensation in ASD patients (132) and may be effective for children's language development (133). At the same time, there are experiments that have counted cases of Omega-3 fatty acids treatment in patients with ASD, found that there is no high-quality evidence that Omega-3 fatty acid supplementation is effective in improving the core and related symptoms of ASD (134). In order to compensate for the inability of Omega-3 fatty acids alone to treat the core symptoms of ASD, research has explored the combined use of Omega-3 fatty acids and vitamin D (VD) and has revealed that the co-supplementation of Omega-3 fatty acids and vitamin D exerts a positive synergistic impact on the social and behavioral aspects in individuals with ASD (135). Because both Omega-3 fatty acids and VD are crucial for brain development and function, their combined supplementation may offer enhanced benefits for cognitive and behavioral health, particularly in populations such as those with ASD, where these nutrients are often found to be deficient or where their metabolic pathways may be altered, the co-supplementation of Omega-3 fatty acids and VD is a potentially safe and effective treatment strategy for the core symptoms of ASD (136). One trial found that treatment with 200 IU/day of VD and 722 mg of DHA significantly improved irritability in children with ASD (137). Based on this ratio, another study confirmed that it can improve symptoms and be able to regulate inflammation levels (91). It has also been shown to improve the senses of people with ASD (138).

The above studies have shown that premature infants are more likely to develop ASD, so some studies have found that Omega-3 fatty acids supplementation during pregnancy can reduce the probability of ASD, and the use of more fish oil can effectively improve the occurrence of the disease (139). EPA/DHA was found to reduce IL-2 levels by measuring red blood cell FAs and cytokines IL-1β and IFNγ in ASD patients (66, 140). Another study found that increased DHA levels and decreased EPA levels were detected in red blood cells in the ASD group compared to the reference group, and the ASD group receiving DHA-rich products had a significant increase in plasma and DHA red blood cell percentages, but no better anti-inflammatory or fatty acid status was found (68). Hence, findings from studies that evaluated the comparative benefits of EPA vs. DHA in enhancing outcomes for ASD patients suggest that EPA could be superior to DHA in the alleviation of depressive symptoms (141). However, many studies have finally confirmed that Omega-3 fatty acids for the treatment of ASD are mostly concentrated in children under 8 years old, and is more effective for their sensory, plasma, and behavioral deficits (142).

### 5.3 Tourette syndrome

TS syndrome is a form of involuntary, repetitive, rapid motor or vocal tics that usually occur during the teenage years, and the exact cause is unknown (143). Research indicates that genetic, neurobiological, and environmental elements may contribute, however, no conclusive therapy has been established (144), only medication-assisted treatment. A double-blind, placebo-controlled trial investigating the effects of Omega-3 fatty acids in the treatment of children and adolescents with TS found that omega-3s may help reduce Tic related damage in some children and adolescents with TS after 20 weeks of treatment with Omega-3 fatty acids in 33 children and adolescents aged 6 to 18 years (145). Another related study, also a double-blind, placebo-controlled trial, also looked at the use of Omega-3 fatty acids in the treatment of TS, further highlighting the potential role of Omega-3 fatty acids in the treatment of TD (146). However, there are few studies on the treatment of TS with Omega-3 fatty acids, and most of them are used as supplements with probiotics and vitamins.

### 5.4 Epilepsy

Epilepsy is a persistent neurological condition primarily characterized by periodic episodes of seizures (147), which are triggered by irregular electrical activity among brain cells (148). Patients may also experience fatigue, headaches, and muscle aches during the recovery period (149). For patients with NDDs, the probability of developing epilepsy is higher (7, 150). A triple-blind randomized clinical trial examining the impact of Omega-3 fatty acid supplementation on clinical and subclinical features in individuals with refractory epilepsy revealed that the supplementation led to considerable decreases in TNF-α and IL-6 levels. Nonetheless, additional studies are required to evaluate various Omega-3 FA constituents for more targeted therapeutic approaches in the treatment of epilepsy (151). In another study exploring the treatment of epilepsy, participants were administered a combination of EPA, DHA, and α-linolenic acid (at a total of 5 g per day) in one arm of the study, and EPA alone (at a dose of 565 mg per day) in another. The research revealed a notable positive correlation between Omega-3 fatty acids and a reduction in seizures; however, the efficacy of this treatment was reported to be low (152). Therefore, the researchers turned to the adjuvant treatment of epilepsy with Omega-3 fatty acids, and found that it can play a potential role in epilepsy in the adjuvant treatment of epilepsy along with anticonvulsant drugs. Furthermore, the use of Omega-3 fatty acid supplementation at a dose of 1,500 mg or below has been associated with a statistically significant decrease in the frequency of seizures, and Omega-3 interventions had a greater effect on adults than in children with epilepsy (153). Simultaneously, the anticonvulsant properties of Omega-3 fatty acids have been documented in animal studies, where they have been observed to increase the latency period before seizures occur and/or elevate the seizure threshold, making it more difficult for seizures to be triggered (154). There are also studies that have found that Omega-3 fatty acids supplementation offers the potential to support the clinical management of drug-resistant epilepsy (DRE) between 0.3 and 1.7 g/day (155). In conclusion, Omega-3 fatty acids have a certain alleviating effect on the treatment of epilepsy, but most of them are only used as supplements for adjuvant treatment, and cannot replace drugs for long-term, targeted and effective treatment (Table 3).

**Table 3:** Comparison of the regulatory mechanism and clinical effects of omega-3 fatty acids in different neurodevelopmental disorders and epilepsy.

**Disease**	**The main regulatory mechanism**	**Clinical effects**	**Research status and challenges**	**References**
ADHD	(1) Regulates neurotransmitter function (dopamine, norepinephrine) (2) Anti-inflammatory effect (lowers IL-6, TNF-α) (3) Improve the fatty acid composition of red blood cell membrane (increase EPA/DHA level) (4) Inflammation may be modulated through the gut-brain axis (GBA).	(1) may improve symptoms of inattention and hyperactivity (short- to medium-term effect) (2) combination methamphetamine (MPH) may improve medication adherence (3) supplementation with GLA (γ-linolenic acid) may be synergistic anti-inflammatory	(1) There are individual differences in efficacy (related to FADS1/2 gene polymorphisms) (2) high-dose EPA/DHA was not significantly superior to placebo (3) larger studies were needed to validate individualized regimens (e.g., genotyping guidance doses)	(121–130)
ASD	(1) Regulates neuroinflammation (lowers IL-1β, IFNγ) (2) Promotes brain development (DHA's role in synaptic plasticity) (3) Combines vitamin D to enhance cognitive and behavioral improvement (4) May improve sensory function (smell, touch)	(1) Improve stereotyped behaviors and social interactions (the effect is more significant in children <8 years old) (2) Vitamin D combined with vitamin D may alleviate irritability (3) Supplementation in preterm infants may reduce the risk of ASD	(1) There was limited improvement in core symptoms with use alone (2) EPA may be superior to DHA in relieving depressive symptoms (3) the small sample size led to uncertainty in the conclusions	(131–138)
TS	(1) Potential modulation of basal ganglia neurotransmitters (Dopamine/5-HT Balanced) (2) Anti-inflammatory effect (the mechanism is not clear)	(1) 20-week supplementation may reduce tic frequency and severity (2) currently only as adjunctive therapy with probiotics or vitamins	(1) Studies were minimal, small trials and (2) long-term efficacy data were lacking	(144–146)
Epilepsy	(1) Decreased pro-inflammatory factors (TNF-α, IL-6) (2) modulated neuronal excitability (increased GABAergic activity) (3) Shown to increase seizure threshold in animal models (4) Possible neuronal protection by inhibiting lipid peroxidation	(1) Adjuvant antiepileptic drugs reduced the frequency of drug-resistant seizures (more pronounced in adults) (2) statistically significant at doses ≤ 1.5 g/day (3) animal studies showed a prolonged seizure latency	(1) Low efficacy of monotherapy (requires combination therapy) (2) Weak effect in children (3) The optimal proportion of ingredients is not clear	(151–155)

## 6 Deficiencies and prospects of current research

Current Omega-3 interventions for ADHD, ASD, TS, and epilepsy show promising yet limited therapeutic effects, constrained by variable individual responses, modest effect sizes, and incomplete mechanistic understanding. To address these challenges, future research must adopt a dual focus on precision medicine approaches and combination therapy optimization. Emerging evidence highlights the critical role of genetic factors like FADS1/2 polymorphisms in determining Omega-3 metabolism efficiency, suggesting the need for genotype-stratified clinical trials to establish personalized dosing regimens. Concurrently, biomarker-driven strategies incorporating erythrocyte membrane EPA/DHA levels and oxidative stress markers could enable dynamic treatment adjustments. Disease-specific EPA/DHA ratio optimization also warrants investigation, particularly for ADHD (high EPA), ASD (balanced ratios with phospholipid carriers), and epilepsy (DHA-rich formulations), while accounting for individual variations in drug interactions and seizure thresholds.

The therapeutic potential of Omega-3 may be significantly enhanced through strategic combinations with adjuvants like VD, probiotics, and antiepileptic drugs, necessitating mechanistic studies to elucidate their synergistic actions on neuroinflammation, gut-brain axis modulation, and GABAergic function. Advanced delivery systems, including nanoemulsions and time-release formulations, could improve bioavailability in high-risk populations such as the elderly and IBD patients. Ultimately, integrating these approaches through predictive modeling and real-time monitoring technologies will be crucial for developing truly personalized intervention paradigms that maximize clinical outcomes across neurodevelopmental and neurological disorders. This comprehensive strategy promises to transform Omega-3 supplementation from a generalized approach to a precision therapeutic tool.

## 7 Conclusion

Omega-3 fatty acids play a vital role in maintaining brain health, regulating inflammation, and supporting the cardiovascular system. In recent years, its multi-pathway regulatory role in the treatment of neurodevelopmental disorders (NDD) and epilepsy has attracted extensive attention. Its mechanism of action covers four core pathways: (1) significantly reduces neuroinflammation by inhibiting the release of pro-inflammatory factors and promoting the production of anti-inflammatory mediators; (2) promote interneuronal connections and information transmission by increasing the expression of synaptic plasticity-related proteins; (3) scavenging free radicals and activating the Nrf2/ARE antioxidant pathway to protect neurons from oxidative stress; (4) by regulating the gut-brain axis in both directions, it can improve the neurotransmitter disorder caused by intestinal flora imbalance and indirectly regulate brain function. This multi-target synergy provides a potential therapeutic window for NDDs (e.g., ASD, ADHD) and refractory epilepsy.

However, the bioavailability of Omega-3 fatty acids is affected by the source (deep-sea fish, algae, etc.), dosage form (ethyl ester form, triglyceride form) and individual metabolic differences, and it is necessary to develop a precision delivery system to optimize their application strategies. As a natural nutrient, omega-3 fatty acids have shown unique advantages in the prevention and adjuvant treatment of neurological diseases. However, due to the current general intake of dietary intake, especially EPA and DHA, there is an urgent need to promote functional food innovation. For example, the development of high-stability Omega-3-fortified dairy products through microencapsulation or the cultivation of high-content algae raw materials using gene editing technology can improve the convenience of ingestion. At the same time, it is necessary to strengthen public education and combine wearable devices to monitor personal nutritional status to achieve personalized supplementation programs.

Future research should focus on the development of multi-compound functional foods by combining plant-derived Omega-3s with other neuroprotective phytochemicals, such as ginkgo biloba extract or green tea catechins. This combined approach amplifies the therapeutic effects while being in line with a sustainable and plant-centric dietary paradigm. In the future, with the integration of multi-omics technology and artificial intelligence, the molecular mechanism map of Omega-3 fatty acids will be further improved. It is expected to develop into a cornerstone treatment under the framework of integrative medicine, aiming to improve the health of patients with neurological disorders.
